# Editorial: Current Perspectives, Challenges and Advances in Cell Based Therapies

**DOI:** 10.3389/fonc.2019.01061

**Published:** 2020-02-06

**Authors:** Monica S. Thakar, Conrad Russell Cruz, Prashant Trikha

**Affiliations:** ^1^Clinical Research Division, Fred Hutchinson Cancer Research Center and Department of Pediatrics, University of Washington, Seattle, WA, United States; ^2^Children's National Health System, Washington, DC, United States; ^3^Center for Childhood Cancer and Blood Diseases, Nationwide Children's Hospital, Columbus, OH, United States

**Keywords:** immunotherapy, NK cell, tumor micoenvironment, CAR (chimeric antigen receptor) T cells, cell based therapy

“Current Perspectives, Challenges and Advances in Cell Based Therapies,” a special collection in Frontiers of Oncology/Immunology, focuses on new developments in the field of cellular immunotherapy. In this collection, we hope to capture the challenges of developing cellular therapy for different diseases, the emergence of new technologies, and the ways cell and immune based therapies can be made safer and more effective. Many of the newest cell-based immunotherapies in the clinic today have focused on chimeric antigen receptor (CAR)-T cells. These therapies have led to impressive clinical results for a subset of diseases with historically poor outcomes. However, while T cells have been effective at eradicating hematological malignancies (most specifically lymphoid diseases), they have so far had limited efficacy against solid tumors (1). While multiple groups continue work on enhancing efficacy of CAR-T cells for solid tumors, several laboratories have begun work on other immune cells. In addition to T cells, innate immune cells, especially natural killer (NK) cells, play a pivotal role at not only eradicating cancer cells but also in modulating the function of adaptive immune cells (2). These cells are emerging as important players in cellular immunotherapy. Concurrently, we have improved our knowledge of the role of the tumor microenvironment (TME) in the pathogenesis of cancer (3). This has increased our understanding of the interaction between cancer cells and immune cells, and the mechanisms by which cancer cells suppress the anti-tumor function of innate and adaptive immune cells.

Several challenges remain, which, when addressed, would dramatically improve efficacy of cell-based therapies, particularly against solid cancers. These include (1) identification of optimal tumor antigens, (2) enhancing trafficking of adoptively transferred cells to tumor and metastatic sites, and (3) neutralizing the immunosuppressive TME. Some of these challenges can be addressed by genetic modification of T/NK cells. Moreover, better characterization of immune cells using mass cytometry (CYTOF) and single cell RNA sequencing will increase our knowledge about their vast repertoire of receptors and genes, which will help decipher their immune functions. In turn, precise augmentation or inhibition of these receptors can potentially make cellular immunotherapy more effective. For example, knocking down inhibitory receptors on cells using CRISPR can augment their anti-tumor function.

We have come a long way since the early days of cell-based therapies, and still exciting new avenues are being explored (4). The development of new technologies to measure metabolic profiles now allows us to interrogate how cellular metabolism regulates immune cell physiology, and how this metabolism impacts the anti-tumor response of immune cells. There are now studies that investigate the fate of immune cells following infusion. This knowledge could then be applied to improving *in vivo* trafficking of effector and regulatory cells to the tumor and metastatic sites. Additionally, toxicities and side effects that develop following the infusion of CAR cells are being better understood, leading to improved strategies that focus on decreasing risks associated with this therapy. Finally, long term effects of these genetically modified cells can now be addressed.

It is our hope that we capture these ideas in the following articles in this collection. While they are understandably not all-encompassing, these articles are representative of current efforts in the field.

Du and Wei explore the role of NK cell immunotherapy in patients with gastric cancers, which the application of emerging immunotherapies to a set of diseases that has not typically been included in immune cellular-based strategies. Chen and Gao study the potential of using anti-LMP CAR-modified T cells against LMP^+^ nasopharyngeal carcinoma. Nayyar et al. provide a detailed overview of challenges seen in using NK cell immunotherapy for solid tumors and provide a systematic overview of methods to improve NK cell function and potential. Ali et al. reviewed the development of CAR T cell therapies for pancreatic cancer, noting the progress in the field and current challenges in utilizing this therapy in this disease. Dwyer et al. talk about common gamma chain cytokines and their role in T cell survival and generation of memory, and how this understanding can pave the way for enhancing cell-based immunotherapies. Shah et al. review the concept of cancer immune evasion and the strategy of targeting multiple antigens as a method to overcome resistance in CAR-T therapies. Liu et al. investigate using CRISPR technology as an engineering method to create a potent and potentially universal CAR-T therapy. Patel et al. summarize non-CAR T gene-modified cell-based approaches—both T cells modified with other transgenes and non-T cell-based therapies. Qin et al. focus on their lab's specific pre-clinical studies evaluating the improved activation of cytotoxic T lymphocytes using an immortalized mouse embryonic cell line (NIH3T3)-conditioned medium as a method to augment adoptive cell therapy. Han et al. examine the mechanisms involved in regulatory T cell modulation of T cell function, looking at avenues to overcome them to enhance anti-tumor immunity. Xu et al. discuss methods to improve CAR T cells by looking more closely into metabolism, effectively improving the function of CAR T cell-based approaches. Wang et al. review innate immune cell-based therapies for osteosarcoma and enumerate strategies to enhance their efficacy. Thakar et al. reviewed the methods to improve the safety profile of CAR-based therapies, describing both pharmacological treatments and signaling pathways involved in cytokine release syndrome.

We anticipate that these articles will provide a state-of-the-art overview of the diversity and challenges faced in this growing arena, and open additional areas of future investigation.

## Author Contributions

All authors listed have made a substantial, direct and intellectual contribution to the work, and approved it for publication.

### Conflict of Interest

CC is a co-founder of Mana Therapeutics, a biotechnology company that is developing cell therapies for cancer. The remaining authors declare that the research was conducted in the absence of any commercial or financial relationships that could be construed as a potential conflict of interest.
